# Real-Time Detection of Railway Track Component via One-Stage Deep Learning Networks

**DOI:** 10.3390/s20154325

**Published:** 2020-08-03

**Authors:** Tiange Wang, Fangfang Yang, Kwok-Leung Tsui

**Affiliations:** School of Data Science, City University of Hong Kong, Hong Kong 999077, China; tgwang2-c@my.cityu.edu.hk (T.W.); kltsui@cityu.edu.hk (K.-L.T.)

**Keywords:** deep learning, railway component detection, real-time, you only look once

## Abstract

Railway inspection has always been a critical task to guarantee the safety of the railway transportation. The development of deep learning technologies brings new breakthroughs in the accuracy and speed of image-based railway inspection application. In this work, a series of one-stage deep learning approaches, which are fast and accurate at the same time, are proposed to inspect the key components of railway track including rail, bolt, and clip. The inspection results show that the enhanced model, the second version of you only look once (YOLOv2), presents the best component detection performance with 93% mean average precision (mAP) at 35 image per second (IPS), whereas the feature pyramid network (FPN) based model provides a smaller mAP and much longer inference time. Besides, the detection performances of more deep learning approaches are evaluated under varying input sizes, where larger input size usually improves the detection accuracy but results in a longer inference time. Overall, the YOLO series models could achieve faster speed under the same detection accuracy.

## 1. Introduction

**Figure 1:**
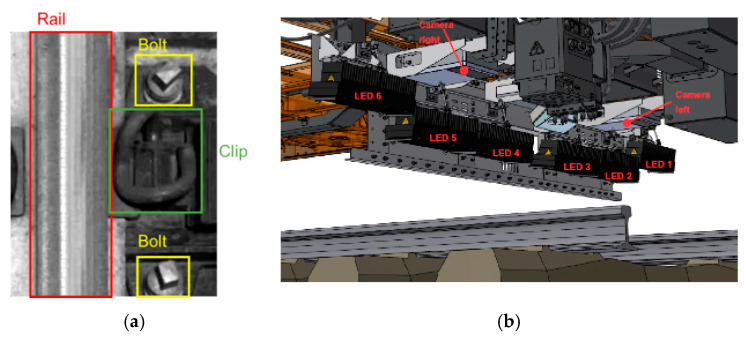
(**a**) Key railway components: rail, bolt, and clip; (**b**) Locations of lights and cameras.

Automatic inspection of railway track has always been attached great significance to the massive construction of railway network. In the research on railway inspection system, many scholars focused on infrastructure systems [1], surface defects detection [2,3], and fastener detection [4,5], etc. To prevent train derailments and other severe accidents due to deficient or lapsed railway components, it is indispensable to perform regular inspection on railway track components including the rails, bolts, and clips (as in Figure 1a), where bolts are used to fasten rail ends together at a joint and the rail elastic clips are used with rail sleeper to fasten rails. Clips and bolts together form fasteners [6]. Traditional methods, either manual inspection or specialized machines, are time-consuming and require skillful technicians [7]. With the continuous expansion of the railway network system, vision-based technologies, which usually take the railway track images as input to extract visual features, have been increasingly popular as they improve the efficiency of component detection work. Aytekin et al. [4] utilized a high-speed laser range finder camera with combination of histogram features and implemented real-time railway fasteners inspection. Xia et al. [8] extracted Haar-like features through railway images and identified the fastener state by the adaptive boosting (AdaBoost) algorithm. Similarly, a combination of support vector machines (SVM) classifier and histogram of oriented gradient (HOG) features were also proposed for railway track inspection [9]. Khan et al. [10] introduced a machine vision-based technique which used Shi-Tomasi and Harris-Stephen features to automatically detect the rail line anchors. Marino et al. [5] presented two multilayer perceptron neural classifiers to process image data from two discrete wavelet transforms to detect the fastening bolts. Li et al. [11] combined evidence from multiple cameras, global positioning system, and distance measurement instrument to robustly detect ties, tie plates, and anchors of railway system. These traditional vision-based methods facilitate the inspection of railway track components with reduced manpower and equipment resources. However, the detection accuracy could easily stagnate because it is hard to manually design accurate and robust feature descriptors for all the railway components due to the diversity of shapes and backgrounds [12].

In recent years, the deep learning methods have attracted much attention from the research world due to the rapid development of graphics processing units (GPUs), which increases the computing power and greatly shortens the training time. The emergence of deep learning methods makes it more achievable to obtain state-of-art results on railway track component detection. Convolutional neural network (CNN), as a category learning method, can map data into nonlinear space and automatically extract potential features, which are more complex and more robust to images containing noises [12]. Some classic CNN models inspired by human visual cortex system, for example, GoogLeNet [13], visual geometry group (VGG) [14], and residual network (ResNet) [15], are originally put forward for image classification. To implement component detection, or more generally, objects detection, the localization and classification of objects are required to label the existing objects with rectangular bounding boxes and also show the confidences of detection. Two types of frameworks are mainly used in generic object detection. One follows two-stage object detection pipeline which includes a region proposal network and a fine-tuned network. The other treats object detection as a regression problem and directly predicts the locations and classes, without the region proposal stage. For two-stage methods, Girshick et al. [16] proposed regions with CNN features (R-CNN), which attained distinct improvements in accuracy of objects detection, and is seen as a major breakthrough in the field of object detection. Subsequently, a series of R-CNN based detection methods have been propound to accelerate the detection speed, including fast R-CNN [17] that finally branches into two output parts: the bounding box predictions and the classifications, faster R-CNN [18] that develops region proposal network to speed up testing. Lin et al. [19] constructed a feature pyramid network (FPN), a generic feature pyramids extractor inside deep convolutional nets that helped detection models to detect objects across a large range of scales. Ye et al. [20] proposed a fusion refine neural network (FR-Net) based on CNN to detect objects ahead in railway shunting mode. FR-Net still applied a two-step strategy: coarse detection module refines locations and prior anchors, finer detection module for more accurate object locations and classifications. Shang et al. [21] applied CNN on cropped rail images to build a two-stage pipeline for localizing and classifying rail defects.

Up till now, the two-stage object detection algorithms have realized high detection accuracy, but the inference time is still intolerable for certain applications, such as real-time pedestrian detection [22] and video analysis [23]. Also, it is hard to optimize each component in this complex pipeline. To address these disadvantages, faster object detection models, such as single shot multibox detector (SSD) [24], RetinaNet [25], and you only look once (YOLO) [26] are proposed. These detection models are called “one-stage” approaches because they unified the region proposal, feature extraction, and classification regression into one deep network. The YOLO model is the very first attempt to build a real-time object detector. As YOLO only passes through the network once to get the predictions, it can upgrade the speed of detection and do inference fast [26]. SSD is one of the first attempts using FPN in CNN for efficient object detection of various sizes. Different from YOLO, SSD utilizes anchors to predict bounding boxes, which is much easier than directly predicting bounding boxes from scratch. Also with the application of anchor boxes, YOLOv2 [27] has been put forward as an enhanced version of YOLO. It provides a solution to small object detection problems with a much larger number of object categories by combining high resolution features and low-resolution features. Additionally, YOLOv2 implements multiscale training to adapt to various image sizes. Later on, YOLOv3 [28] was put forward with “more advanced” design tricks containing multiscale prediction output and more complex feature extractor. Yanan et al. [29] took advantage of YOLOv3 algorithm to realize rail surface defect detection. The results were shown as accurate and fast. The inference time of YOLOv3 increased because of large number of layers. To raise the inference speed and make sure detection accuracy at the same time, the partial residual network based YOLOv3 model (YOLOv3-PRN) was then initiated [30]. Lin et al. [25] also introduced the RetinaNet as a one-stage dense object detector which makes use of FPN and focal loss as two crucial building blocks.

So far, the one-stage detection models have achieved great success on classic image datasets, but few of them were put into applications on specific railway detection tasks. This paper focuses on railway track component detection based on YOLO family models. Different from the above works applied to large and general datasets, our model focuses on small railway track image dataset. By optimizing the parameters and comparing between other deep learning models, our work can obtain the best detection performance on railway track component detection in a limited dataset. The railway track images utilized in this study are provided by the Hong Kong Metro Corporation (MTR). Figure 1b displays the image acquisition system used for image collection. Two cameras and six LED lights are installed under the train. Because of the vibration when the train is running, images are taken from different angles and under different illumination conditions. The detection performance of models is evaluated in terms of detection accuracy, detection speed, and robustness against varying input resolutions.

The major contributions of this work are summarized as follows:

- To improve object detection results, we adjusted the detection layers of one-stage network to enhance the performance and optimize the parameters. The different size of anchors and inputs are also discussed to make the conclusion more reasonable.
- According to experiments, the advantages and limitations of the state-of-art YOLO models are revealed. The YOLOv3 shows no advantage in the component detection even it has finer feature extraction and more complicated structure of network.
- This paper provides a novel view of the research field, as it differs from existing papers in terms of the specific application on railway component detection. The success of representative one-stage models on railway track components detection can offer practical guidance on other industrial detection tasks such as track defects diagnosis.

## 2. Methodology

The models used in railway track component detection are all one-shot detection models which require only a single pass through the neural network and predicts all the bounding boxes in one go. In this section, some representative one-stage detectors such as YOLO, YOLOv2, and YOLOv3 are introduced. Based on the YOLO series models, we proposed YOLOv2 and YOLOv3 with anchors adjusted to batter adapt to our dataset.

### 2.1. YOLO Series

**Figure 2:**
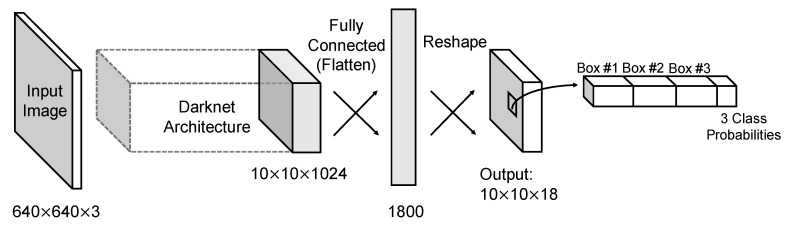
Architecture of YOLO model. The output is a S × S × (5 × B + C) tensor corresponding to the categories, locations, and sizes of objects.

The YOLO model is built on the basis of Darknet which uses a modified GoogLeNet as the backbone. Darknet follows the general design of 3 × 3 filters to double the number of channels at each pooling layer and 1 × 1 filter to periodically compress the feature representation throughout the network. Most of the convolution layers have been pre-trained with the ImageNet dataset before adapted to the detection task. After 24 convolution layers, 2 fully connected layers are followed to integrate the predictions. YOLO algorithm simply splits image into fixed S × S cells and each cell predicts fixed B bounding boxes. So the output of final layer is a S × S ×(5× B + C) tensor corresponding to the predictions of bounding boxes, where C is the number of classes that need to be estimated and 5 is related to four coordinates with one confidence value. The coordinates of bounding box are defined by a tuple of 4 values: center x, center y, width w, and height h. The confidence score which represents “objectness” indicates the likelihood of the cell containing an object: $\mathrm{Pr}\left(\mathrm{Object}\right)\times {\;\mathrm{IoU}}_{\mathrm{pred}}^{\mathrm{truth}}$, where Pr(Object) is the probability whether the cell contains an object and IoU represents interaction over union between the predicted box and the true labeling box. In the training process, if the center of object falls into a cell, the one bounding box which has highest IoU out of B bounding box candidates is the “responsible” predictor for predicting probabilities of this object belonging to every ${\mathrm{Class}}_{i}$, that is, $\mathrm{Pr}\left({\mathrm{Class}}_{i}|\mathrm{Object}\right)$. Basically, class prediction is performed at the grid cell level. The only set of C conditional class probabilities given by each cell are shared between B bounding boxes, which greatly limits the capability of YOLO to detect dense objects of different classes. This was later revised in YOLOv2 to generate class probabilities for each bounding box by using softmax activation and cross entropy loss. Figure 2 shows the basic architecture of YOLO model in this paper. The input image size is raised to 640 × 640. With 1/64 downscaling rate, images are divided into 10 × 10 grid. In each grid cell, 3 bounding boxes are predicted and share the same set of class probabilities. By integrating classification and detection together, only a single pass through the neural network is required and it will save time efficiently in the inference process. The loss function in YOLO model consists of two parts, the localization loss for bounding box coordinates prediction and the classification loss for conditional class probabilities. Both are computed as the sum of squared errors. In testing process, the non-maximum suppression (NMS) method [31] is applied to get rid of redundant bounding boxes.

YOLOv2 is an enhanced version of the basic YOLO model. It adopts the concept of anchor priors in SSD. Rather than expecting the model to directly predicted bounding box descriptors, a collection of anchor boxes with varying aspect ratios that indicate some prior information about the size of objects are defined. Except for the improvement on anchors, YOLOv2 also randomly resized every 10 batches to make multiscale training possible in YOLOv2, which helped to train the model to be robust to images of different sizes. Another essential change is that YOLOv2 had less layers than the original YOLO, with only 19 convolution layers composed of 3 × 3 and 1 × 1 filter. Max pooling layers following each convolution layer are for reducing the feature map dimension. Instead of fully connected layers, YOLOv2 applies route layer and reorg layer on the feature maps to get fixed dimension of output. The route layer is to bring finer grained features in the network from earlier; the reorg layer is to make these features match the feature map size at the later layer.

YOLOv3 adds a bunch of design tricks inspired by latest advances in the object detection field. First of all, the new Darknet-53 architecture still relies on successive 3 × 3 and 1 × 1 filters but has residual blocks inspired from ResNet [15] added. Secondly, additional convolution layers follow the base feature extractor to predict bounding boxes at three different levels. This allows YOLOv3 to take advantage of finer-grained information from earlier route layers to further enhance small objects detection. Moreover, contrasted with YOLOv2, YOLOv3 increases the shortcut layers to enlarge the whole network. Reorg layers are replaced by up-sample layers in YOLOv3 to increase the size of feature map. In addition to these changes, multiple independent logistic classifiers replace the soft-max layer to predict class score, which is helpful especially when one image might have multiple labels but not all the labels are guaranteed to be mutually exclusive. Although concatenation has existed in the second version, it becomes more frequent in YOLOv3 that both coarse-grained feature map and fine-grained feature map are provided to realize both large and small objects detections. The result of these concatenations is the formation of three output layers with a down-sampling rate of 1/32, 1/16, and 1/8, respectively.

### 2.2. Our Proposal

**Figure 3:**
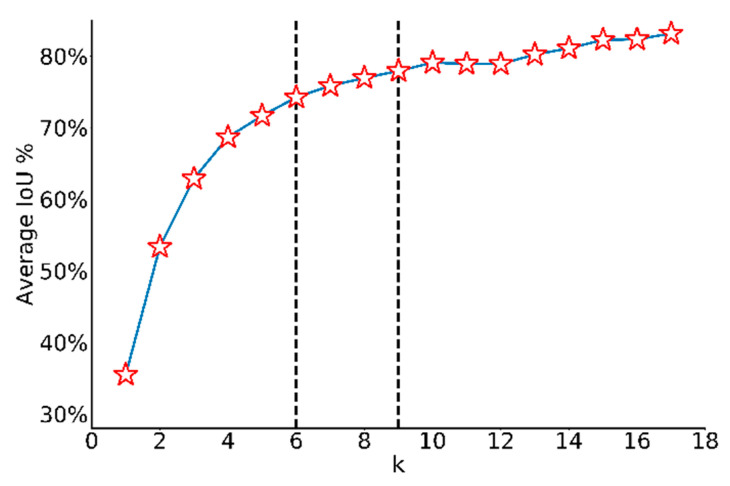
Average IoU under different k values. With k increasing, the average IoU increases.

With the dataset changed to railway track images, *k*-means clustering method was adopted on discovering the best aspect ratios of prior boxes with the distance metric changed to 1-IoU. Since the number of clusters *k* needs to be chosen according to manual experience, average IoU between ground truth boxes and the closest centroid under different *k* is calculated in Figure 3. It is reasonable that with *k* increasing, the average IoU increases because of more prior choices. Considering the trade-off between model complexity and difficulty of predicting, 6 anchors are chosen for YOLOv2 and 9 anchors are set for YOLOv3. With these anchors, coordinates are predicted as the offsets of the center of anchor boxes, widths and heights are predicted as the scale of anchor boxes, which is much easier than directly predicting from scratch in YOLO model. Plus, predicted bounding boxes will not share the same set of classification probabilities anymore, meaning that one grid cell is capable to detect multiclass objects.

**Figure 4:**
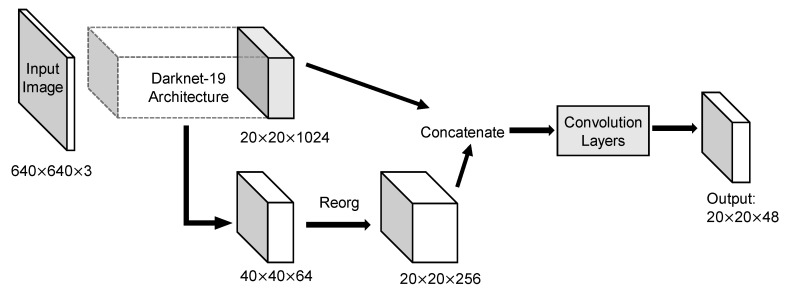
Architecture of YOLOv2 model. Concatenation of coarse-grained feature map and fine-grained feature map helps to combine more features to improve detection. With anchors set to 6, the output became larger than YOLOv2.

**Figure 5:**
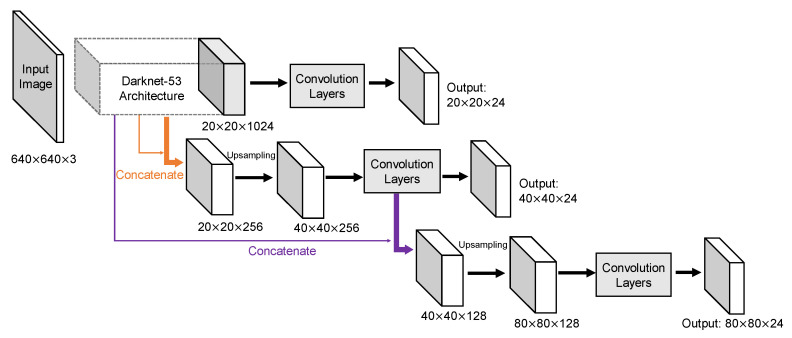
Architecture of YOLOv3 model. Pyramid network leads to a greater output but also helps to extract more potential features. With anchors set to 9, each layer had 3 anchors sort descending.

The YOLOv2 architecture in detail was displayed in Figure 4. Input image size for training and testing are both 640 × 640. Because convolution layers of YOLOv2 down-sample the input dimension by a factor of 32 and the number of anchors is 6, the output tensor should be 20 × 20 × 48. Route layer brings higher resolution feature map 40 × 40 to pass through reorg layer so that it can be concatenated with other feature maps at 20 × 20 resolution. Since the fine-grained feature maps in YOLOv2 are just split across multiple channels, not as outputs, a more standard pyramid output structure has been improved in the third version. Figure 5 intuitively explains the architecture of YOLOv3. With a multiscale output, the model must deal with more bounding box candidates of various sizes. From Figure 3, 9 anchors calculated by *k*-means clustering method are assigned to three resolution output with anchor sizes sort descending. The inference time of YOLOv3 slows down because of more predictive quantities. Nevertheless, it could achieve a high enough detection accuracy.

Since these detectors have images as input, their application areas are quite wide. The only difference is that such a model does not guarantee excellent performance in every data set. For a specific data set, the model structure and parameters need to be adjusted and optimized to achieve the best detection effect. This is also one of the focuses of our work.

## 3. Data Collection

**Figure 6:**
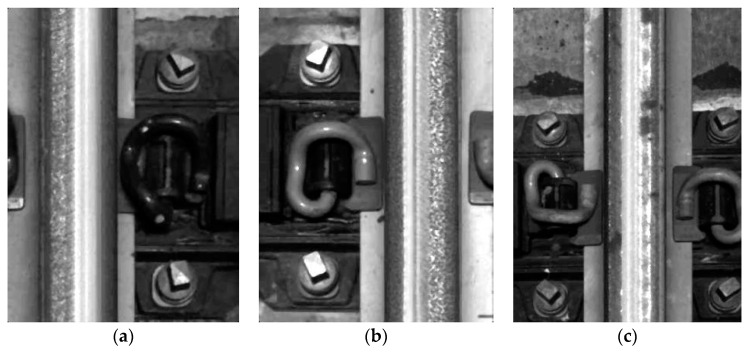
Railway track images from different angles: (**a**) Left side; (**b**) Right side; (**c**) Middle way.

**Figure 7:**
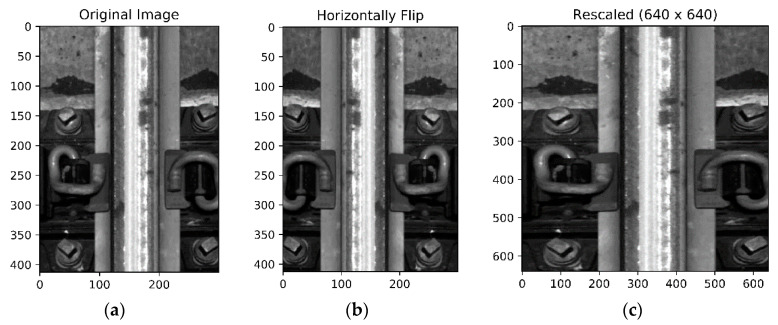

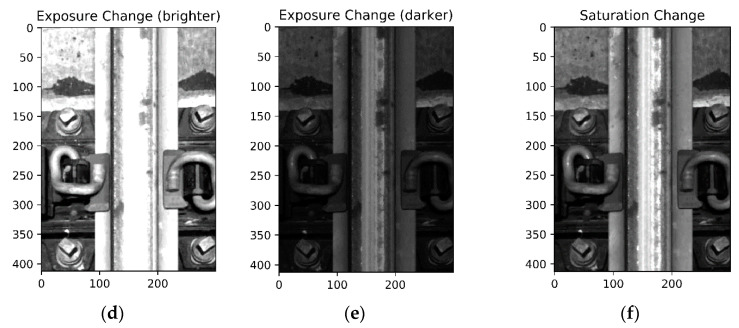
Samples of data augmentation: (**a**) The raw image captured by the image acquisition system; (**b**) The railway track image was flipped from left to right; (**c**) The railway track image was resized to 640 × 640; (**d**–**f**) Different from the geometric distortions above, the photometric distortions include the exposure and saturation change.

The rail surface images are captured with an image acquisition system that cameras are installed at the bottom of the train. Figure 6 shows the image samples captured by the image acquisition system. Due to the vibration when the train is running, pictures are taken from different angles and under different illumination conditions, the size of the captured images also varies a lot. In total, 1386 railway images are manually labeled, where class number, center coordinates, and bounding box sizes are collected as the ground truth labels of corresponding objects. Because of different camera angles, some objects are partially captured and locate at the very edge of the image. In this case, they have smaller bounding boxes as the true label information. In order to make the predicted widths and heights unaffected by image sizes, all the coordinates and sizes of labeling boxes are normalized. The whole dataset is then randomly split into training, validation, and test data sets with a ratio of 2:1:1 for further evaluation. Table 1 summarizes the labeling information. Basically, each image includes at least one rail track with varying number of bolts and clips. Our currently available dataset on railway track images is quite small compared to the public domain dataset such as Pascal VOC dataset and COCO dataset, both of which consist of more than 10 thousand well annotated images for training. However, compared to the detection task with large amounts of images captured under identical conditions, the railway component detection with limited dataset under diverse conditions are much more challenging. To reduce overfitting as much as possible, artificially enlarging the dataset used in training is essential. Some data augmentation methods such as mirror flipping, exposure change, and saturation change [32] are applied to increase the training volume. Figure 7 displays the samples of data augmentation. Considering the railway track should always be vertical, any angle change may damage the detection performance, we just flip images horizontally (we tried to rotate images by 90 degree, the result is a disaster). Also, in the training process, images are randomly rescaled into different sizes to increase the robustness of detection model. In the testing process, images are rescaled outward and inward. While scaling outward, the final image size will be larger than the original image size (the new scale is manually set, we have tried different testing scale, like 640 × 640, 448 × 448, 256 × 256, and 128 × 128). Except for that, other augmentation parameters such as saturation exposure are set to increase the variety of lighting level.

## 4. Training and Testing

### 4.1. Training Setup

The experiments are carried out on our lab server (OS: Ubuntu 16.04, RAM: 32 GB, GPU: Nvidia GeForce GTX 1080 Ti). In the training process, loss value gradually decreases as the number of iterations increases. The maximum number of iterations is set to 10,000 to make sure enough training. For each iteration, 64 images are fed as the input to learn. The learning rate is initialized at 0.005 because of the small training volume, and then multiplied by 0.1 at iteration 8000 and 9000, respectively. For fair comparison, the sizes of input images for all three models are raised to 640 × 640. When the widths and heights are changed at pixel levels, interpolation of pixel values is applied to stretch or compress images. The YOLO model directly predicts the coordinates and sizes of bounding boxes, whereas the YOLOv2 and YOLOv3 models employ *k*-means algorithm on normalized labels of training dataset to choose appropriate anchor sizes. According to the clustering results, the average IoU of anchors used in YOLOv2 is 74.22%, in YOLOv3 is 77.91%. As mentioned before, with *k* increasing, average IoU will increase so the clustering of YOLOv2 has less average IoU than YOLOv3. After training, the weights lead to the highest accuracy on validation set will be evaluated on test set.

### 4.2. Performance Metrics

The detection performance is evaluated using three commonly used metrics: mean average precision (mAP), F1 score, and inference time. The mAP and F1 score are defined as following.

(1) $$\mathrm{Precision}=\frac{\mathrm{TP}}{\mathrm{TP}+\mathrm{FP}}$$

(2) $$\mathrm{Recall}=\frac{\mathrm{TP}}{\mathrm{TP}+\mathrm{FN}}$$

(3) $$F1-\mathrm{score}=2\times \frac{\mathrm{Precision}\times \mathrm{Recall}}{\mathrm{Precision}+\mathrm{Recall}}$$

(4) $$\mathrm{AP}=\frac{1}{11}\underset{R\in \left\{0,0.1,\ldots ,1\right\}}{\sum }p_{\mathrm{interp}}\left(r\right)\times 100\%$$

(5) $$p_{\mathrm{interp}}\left(r\right)=\underset{\tilde{r}:\tilde{r}\geq r}{\underbrace{\max}}p\left(\tilde{r}\right)$$

(6) $$\mathrm{mAP}=\frac{1}{\mathrm{number}\;\mathrm{of}\;\mathrm{classes}}\;\mathrm{AP}$$

Specifically, mAP is the mean of the average precisions (AP) computed over all the three classes (rail, bolt, and clip). The AP metric considers both the spatial position and the accuracy of categories for the detected objects. By setting thresholds, all the predicted boxes with different class scores ($\mathrm{Pr}\left({\mathrm{Class}}_{i}\right)\times {\;\mathrm{IoU}}_{\mathrm{pred}}^{\mathrm{truth}}$) are determined to be whether positive or negative, true or false. Overall, four categories: true positive (TP), true negative (TN), false positive (FP), and false negative (FN) are divided to calculate the precision and recall values before evaluating AP value. Precision is the proportion of true positive examples over all the positive classes that model predicted. Recall is the proportion of true positives over all the unique true examples, which means the value of recall totally depends on the number of true positives because the number of ground truth is fixed. The F1 score is the harmonic mean of precision and recall at certain thresholds. It weighs precision and recall equally which means that the measurement does not emphasize or attenuate the influence of false negatives. Unlike F1 score, which is limited by different thresholds, AP is computed as average value of 11 points on precision/recall curve for each possible recall levels [0, 0.1, …, 1] only under certain IoU threshold. The precision at each recall level *r* is interpolated by taking the maximum precision measured for a method for which the corresponding recall exceeds *r*. In a traditional way, mAP values at a single IoU of 0.50 (${\mathrm{AP}}_{50}$) will be the main metrics to choose final weights models of component detection. Except for detection accuracy, inference time is another important criterion for the model performance evaluation. Inference time, same as image per second (IPS), directly decides whether a detection model is capable to deal with enough images in every second. Although many models pursue “real-time” detection capabilities, it is in fact a pretty vague term. Usually, “real-time” means the algorithm will run at the rate of the source (e.g., a camera) supplying the images, which is producing output simultaneously with the input. There is not a specific frame rate tied to the definition of “real-time”, but undoubtedly, the faster images are processed, the better the algorithm is. Finally, the algorithm complexity is represented by billion float operations (BFLOPs), which is accumulated over multiple convolutions. For a given GPU, the inference time is directly related with BFLOPs.

### 4.3. Results and Analysis

The three one-stage approaches are trained with parameters set in part A. The AP, F1 score, and IPS results of each class for all test samples are summarized in Table 2. The mAP (AP_50_) values of YOLO and YOLOv2 are both above 90%. Although YOLOv3 has more standard pyramid output architecture, it has no advantage on mAP performance of this component detection. YOLOv3 achieves a similar performance with the other two models in bolt and clip detection, however, for rail, the AP value is lower than the other two models. The precision and recall results when threshold is set at 0.25 are also shown in Table 2. Compared to YOLO, YOLOv2 increases the ratio of true positives and results in a greater recall value. However, the number of false positives in YOLOv2 also increases, making YOLOv2 no advantage in the comparison of precision value. YOLOv3 behaves quite different. First, since the unique truth is fixed, the reducing of true positives in YOLOv3 leads to small recall value. Then, false positives must also be less than before so that precision value at 0.25 threshold is much greater in YOLOv3. Both explain that YOLOv3 made efforts on reducing false positives that misclassify negatives as positives. For the inference time, YOLOv2 is the fastest among three models, which is a clear advantage over the original YOLO when both have close mAP performance. The inference speed of YOLOv3 drags down because of the expansion blocks that contain residual connections. If we look at the algorithm complexity, YOLOv3 owns the largest number of convolution operations. Usually a bigger network will take more time to evaluate an input because inference requires propagating the input through all the network to get to the last layer (output). It is worth mentioning that the first two versions of YOLO took less than 24 h to finish all the training process, however, the training time cost with YOLOv3 model is much longer. Table 2 also tabulates the detection results at different IoU thresholds. Except for the mAP at IoU of 0.50, mAPs at IoU of 0.75 (AP_75_), IoU of 0.95 (AP_95_) and averaged over multiple IoU values from 0.50 to 0.95 with a step size of 0.05 (AP_[0.5:0.95]_) are also provided. It’s observed that higher IoU thresholds reward detectors with better localization. Even averaged over IoU, the detection performance of YOLOv2 still outperformed the other two methods. As the IoU threshold increases, the disadvantages of original YOLO model gradually emerge, and the averaged detection results are not as good as YOLOv3.

**Figure 8:**
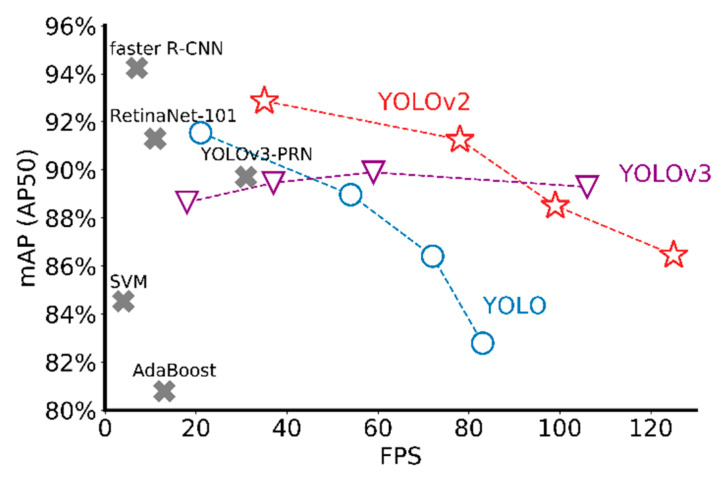
Comparison with other detectors.

To explain the unexpectedly poor performance of YOLOv3 in our results, two potential reasons are given as following. First, compared with the volume of images used for training, the YOLOv3 with more than 100 layers is overcomplicated so that it gradually showed an overfitting trend during the long training process. Second, the design of FPN in YOLOv3 aims to detect small and dense objects. However, the objects in our railway application is not either small or densely located. In fact, during the training process, the last two output layers with more divided grids usually have “invalid” output with no detection results to calculate recall or precision. The three models with various input sizes including 640 × 640, 448 × 448, 256 × 256, and 128 × 128 are trained for better explanation. The mAP and IPS values on the test data set are plotted in Figure 8, where the image size of each model is decreasing from left to right. All three networks can perform detections at different input sizes. The detection speed is faster at a smaller input size. For YOLO and YOLOv2, the detection accuracy decreases as input size decreases. For YOLOv3, large fluctuations in input size cannot affect the mAP value as obviously as the other two. When compared with other detectors including state-of-art deep learning methods and traditional machine learning approaches, faster R-CNN (VGG-16 as the backbone) appears a higher mAP value than the others, but the inference speed is much slower. With region proposal network (RPN), faster R-CNN worked better in terms of detection accuracy because of finer predicted bounding boxes. However, the RPN requires one extra “stage” in the architecture to generate more proper regions for each picture, which directly leads to slower detection speed. As another one-stage detector, RetinaNet-101 behaved close detection accuracy and speed to the first version of YOLO. As for YOLOv3-PRN, a partial residual network based detector, it only speeded up the inference time but maintained the same accuracy. For SVM and AdaBoost, both are machine learning classifiers. By combining with manually designed features such as HOG and Harr-like features, they can implement component detection but not either accurate or fast. Overall, YOLOv2 displays significant advantages in terms of inference accuracy and speed.

### 4.4. Further Experiments

**Figure 9:**
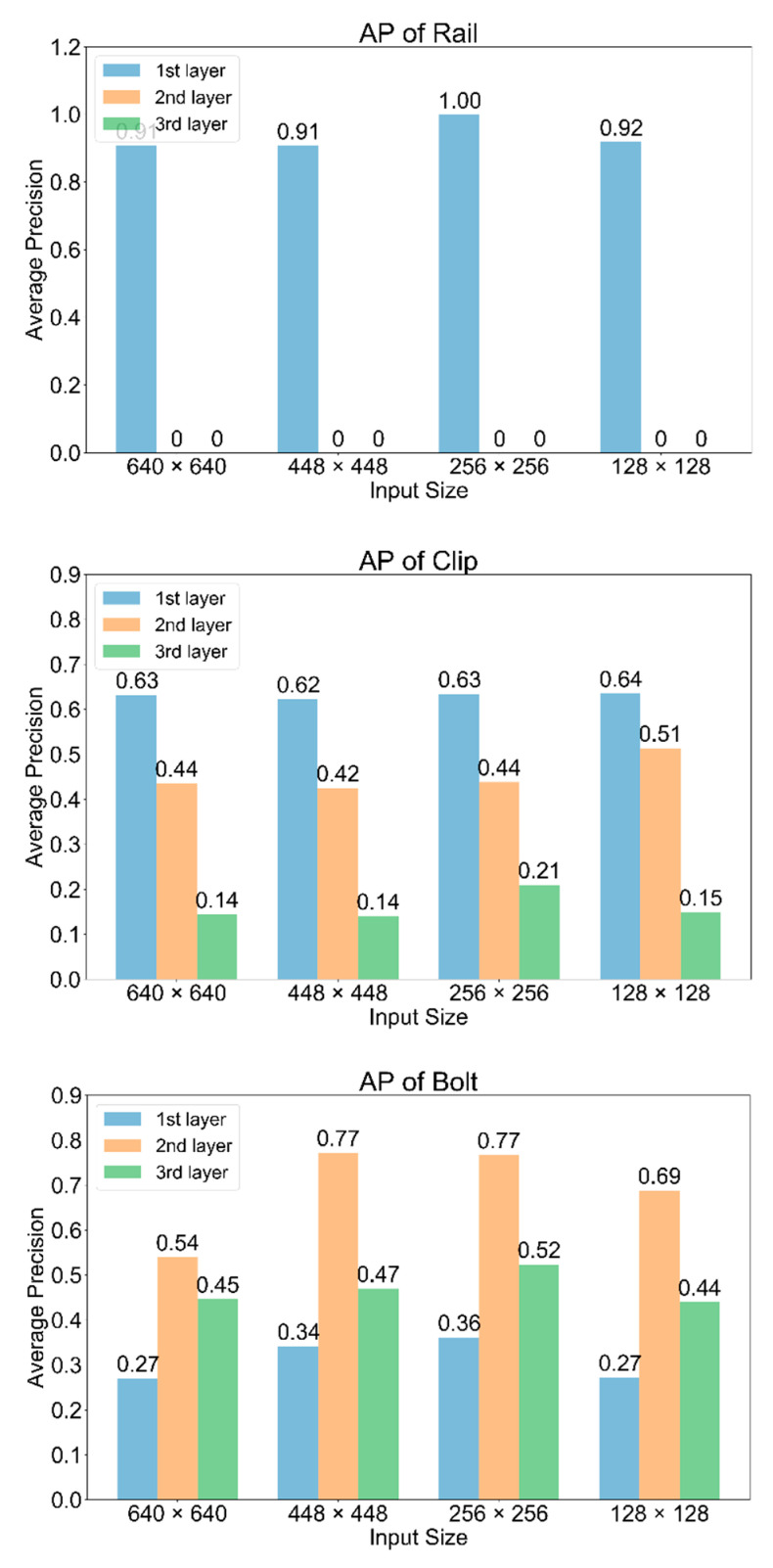
AP values of three classes on different layers with various input sizes.

To further investigate the “abnormal” performance of YOLOv3, we tested mAP on different detection layers under different input sizes. Results are displayed in Figure 9. From the AP plot of rail, only the first layer which outputs a smaller tensor (with larger divided grids) has the rail detection results, while the other two layers that outputs larger tensors cannot detect rails at all. For clip detection, the layer with finer features does not show any advantage. AP values decrease with the output size getting larger. As the smallest component, the detection of bolt mainly happens on the last two layers, and the second layer contributed the most accuracy. Another finding is, no matter with what input size, the first detection layer is always good at large component detection (rail and clip). The other two layers with finer features and more grid divides perform better on bolt detection. Specifically, the second layer detects the bolt more accurately than the last layer, which suggested that blindly enlarging the output is not conductive to bolt detection. On the contrary, it will lead to the occurrence of overfitting and hurt the detection result.

Moreover, to fully understand the effect of anchors on the results, YOLOv2 and YOLOv3 are compared with different anchors. In Table 3, changing anchors barely influenced the mAP (${\mathrm{AP}}_{50}$) but lead to some changes in ${\mathrm{AP}}_{75}$ and ${\mathrm{AP}}_{95}$, which implied that having more anchors would provide better initial information about object size and improve the detection accuracy under a higher IoU threshold. Also, with the number of anchors increasing, IPS became less which suggested the inference time was longer. Considering the YOLOv3 is overfitting during training process, we cut the detection layers to simplify the model. The last two rows in Table 3 show that, with only one and two output layers, the detection accuracy got damaged while the inference speed was efficiently improved. Even the number of anchors increased, there is no sign that YOLOv3 could work better than YOLOv2. The experimental results agree with our previous assumption that YOLOv3 has an overcomplicated architecture this railway track component detection. Although it has a multiscale architecture to extract more potential features, it becomes a burden in this application. Hence, we conclude YOLOv2, with the best accuracy and fastest speed, as a better detection model for real-time railway inspection and further component detection.

## 5. Conclusions

In this work, one-stage deep learning networks including YOLO, YOLOv2, and YOLOv3 are proposed to detect railway track components including rail, bolt, and clip. The detection performance is evaluated through Hong Kong MTR railway surface images with different sizes and illuminations. The YOLO series models can accurately detect almost all the key components in the railway image. Compared to original YOLO model (mAP: 92%, inference time: 21 IPS), the YOLOv2 model improves the performance in both detection accuracy (93% mAP) and inference time (35 IPS) and can detect mostly cropped objects such as clips. In contrast, the YOLOv3 yields the lowest mAP of 89% and takes more detection time than the other two. Besides, all three networks predict detections at different input resolutions. The YOLO and YOLOv2 improve the detection accuracy with larger input sizes but lead to slower inference speed, whereas the YOLOv3 shows slightly better performance at smaller input resolutions and with less anchor boxes. When compared with other one-stage models such as RetinaNet-101 and YOLOv3-PRN, the YOLO series models always show satisfying detection results on both accuracy and speed. To conclude, YOLOv2, with the high accuracy and fastest speed, is the most suitable model for real-time railway inspection and further component detection.

Nevertheless, the results suggest some further applications.

- The parts on both sides of the rail are symmetrical, especially for the images that photographed by the camera directly above the rails. With this, it is hopeful that the missing parts of these components can be detected.
- With advantages in detection accuracy and speed, YOLOv2 has the potential to be applied to video and finer object detection.
- YOLOv3 with fine-grained feature maps has advantage of detecting small objects. Therefore, using YOLOv3 for track defects diagnosis is another direction for railway inspection.

## Figures and Tables

**Table 1:** Labeling information.

Data	Rail	Clip	Bolt
Training set	692	1016	1460
Validation set	352	545	783
Test set	348	512	742

**Table 2:** Performances on testing data set.

Model	IoU = 0.50, Threshold = 0.25										IPS	AP_75_	AP_95_	AP_[0.5:0.95]_	BFLOPs
	mAP	Rail	Clip	Bolt	Precision	Recall	F1	TP	FP	FN					
YOLO	92%	100%	89%	86%	0.89	0.93	0.91	1557	192	123	21	43%	2%	45%	82.0
YOLOv2	93%	100%	90%	89%	0.83	0.97	0.89	1629	339	51	35	53%	3%	50%	69.5
YOLOv3	89%	91%	90%	87%	0.94	0.84	0.88	1404	89	276	18	47%	5%	49%	154.5

**Table 3:** Comparison on anchors and layers.

Model	mAP (AP_50_)	IPS	AP_75_	AP_95_
YOLOv2				
3 anchors	93%	44	47%	0.1%
6 anchors	93%	35	53%	3%
9 anchors	93%	32	53%	3%
12 anchors	93%	29	62%	4%
YOLOv3				
3 anchors	89%	21	42%	0.2%
6 anchors	89%	21	43%	3%
9 anchors	89%	18	47%	5%
12 anchors	87%	13	46%	6%
1 layer	87%	43	49%	0.6%
2 layers	87%	31	45%	0.5%
